# Integrative Techniques for Hybrid Breeding in *Eucalyptus*: Advances, Innovations, Challenges, and Future Prospects

**DOI:** 10.3390/plants15182841

**Published:** 2026-09-17

**Authors:** Mncedisi Ndwandwe, Hussein Shimelis

**Affiliations:** 1 Institute for Commercial Forestry Research, School of Agriculture and Science, University of KwaZulu-Natal, Pietermaritzburg 3201, South Africa; 2 African Centre for Crop Improvement, School of Agriculture and Science, University of KwaZulu-Natal, Pietermaritzburg 3201, South Africa

**Keywords:** hybrid breeding, genetic analysis, genome-assisted selection, market-driven breeding, quantitative trait loci

## Abstract

Hybrid breeding exploits heterosis from genetically complementary parents to develop desirable products, including in *Eucalyptus*. The aim of this review is to present integrative techniques for hybrid breeding in *Eucalyptus* based on conventional methods such as phenotypic selection and controlled hybridization, and modern genomic-assisted approaches to guide product development. Molecular breeding tools such as marker-assisted selection (MAS), quantitative trait loci (QTL) mapping, genotyping-by-sequencing (GBS) and next-generation sequencing (NGS) have enhanced the precision of selection in *Eucalyptus* for traits such as growth rate, wood density, fibre quality, disease resistance, drought tolerance and adaptability. Modern approaches have enabled the identification of trait-specific QTL and genes in popular *Eucalyptus* species such as *Eucalyptus grandis*, *E. urophylla*, *E. camaldulensis*, and *E. nitens*, thereby accelerating hybrid development. Genome editing techniques offer new opportunities for genetic modification of traits, especially in *Eucalyptus*, where the final product may not require stringent regulatory oversight. In *Eucalyptus* breeding, the key market-preferred traits are pulp and paper, lumber, and bioenergy, requiring high-performance and better biomass hybrids. For successful hybrid breeding, a multidisciplinary approach is required that integrates genetic and genomic resources. Furthermore, investment in forest genomics and multi-institutional collaboration will be needed to keep up with advances in *Eucalyptus* breeding. The review may serve as a framework to guide *Eucalyptus* breeding and genetics, aiming to develop next-generation hybrids that adapt to environmental changes and meet market demands.

## 1. Introduction

The demand for wood-based products such as timber and pulp, and biomass for bioenergy, has steadily increased in recent decades, driven by growing populations, urbanisation, increasing infrastructure, and the need for renewable sources worldwide [1]. Wood/biomass products are used in the construction, packaging, and bioenergy industries. The use of high-performing hybrids in the forestry industry is vital in sustainable wood and energy supply systems. To meet this demand sustainably, high-yielding tree species will be required that can be bred and cultivated on a commercial plantation scale for multiple utilities.

*Eucalyptus* is one of the most economically and widely used genera in industrial forestry, with more than 700 species with different ecological adaptations [2]. *Eucalyptus* species originated in Australia and the islands north of Australia and are now grown extensively in tropical, sub-tropical, and temperate regions globally, including South America, Africa, and Southeast Asia. *Eucalyptus* species are a good choice for wood and energy supply because of their rapid growth, adaptability to different climates, and high biomass productivity [2].

Due to their distinctive novel phenotypic attributes and varied product profiles, several *Eucalyptus* species are recognised globally. For instance, *E. grandis* is a fast-growing, erect species which is widely cultivated, serving as a desirable parent in hybrid breeding programmes [3]. Whereas the species *E. urophylla* is popular for its tolerance to common pests and diseases, especially in tropical climates [4]. The species *E. smithii* produces dense wood suitable for the pulp and fuelwood industries. Reportedly, *E. camaldulensis* is known for its drought tolerance and wide adaptation in dryland and arid ecologies [5], while *E. nitens* has exceptional cold tolerance and is adapted to temperate climatic conditions. The above species are unique genetic resources that can be selected as breeding parents based on the breeding goals, growing conditions, and the desired end products.

Each species has its own utility and merit, but the pace of genetic gain through traditional breeding is often limited [6]. Multiple traits are simultaneously selected, e.g., growth rate, wood quality, disease resistance, and environmental tolerance. Intra-specific breeding often fails to combine the full range of traits needed for high-performance *Eucalyptus* populations due to genetic bottlenecks. Selection response for these traits can be enhanced through targeted breeding and modern genomic tools.

Interspecific hybridisation has emerged as a powerful breeding strategy owing to heterosis or hybrid vigour. Crosses of genetically distant and compatible species combine desirable features into a single genotype, which may result in hybrid vigour [7]. For example, hybrids between *E. grandis* and *E. urophylla* are among the most widely deployed due to their exceptional growth and disease resistance [8]. *E*. *grandis* × *E. nitens* hybrids are also mostly deployed because of their exceptional frost tolerance and straight growth forms. Similarly, crosses involving *E. camaldulensis* can enhance drought tolerance, while *E. globulus* hybrids may improve pulp quality [9]. These combinations allow breeders to tailor *Eucalyptus* varieties to meet both ecological and market needs.

There are some limitations in interspecific hybridisation. Controlled cross-pollination between species is technically challenging and time-consuming, and the success rate of hybrid seed production is often low and inconsistent [6]. Also, hybridisation is followed by clonal selection from the segregating generation of *Eucalyptus* hybrids. Clonal propagation is costly and requires extensive infrastructure, such as dedicated nurseries and aseptic propagation systems, thus increasing the costs of breeding programmes [3].

The recent development of marker-assisted selection (MAS), quantitative trait loci (QTL) mapping, and genomic selection is increasing the accuracy and efficiency of conventional breeding methods [9]. For instance, hybrid clones of *Eucalyptus* with better physical properties for plywood production are being explored [10]. Research on inbred progenies and elite clone development is also aiding in the streamlining of hybrid seed production [11]. Biotechnology and genomics are contributing more to optimizing hybrid breeding globally, opening new avenues to increase productivity, disease resistance, and environmental resilience in *Eucalyptus* plantations [12].

There is a need for up-to-date, coherent information that integrates advances, innovations, challenges, and opportunities in hybrid breeding to guide the design and commercialisation of new *Eucalyptus* breeds. This review integrates the advances in classical breeding approaches, including effective pollination and phenotypic selection, with the new genomic-assisted technologies such as MAS, QTL mapping, Genotyping-by-Sequencing (GBS), and next-generation sequencing (NGS). The first section presents genetic resources and breeding approaches for improving *Eucalyptus*, followed by market-driven applications that align with market needs and their influence on breeding priorities. Finally, the review presents the development of modern genotypes, key gene banks and databases, and breeding gains in productivity, resilience, and quality. The review concludes with a discussion of hybrid breeding and future directions. The review may serve as a framework to guide *Eucalyptus* breeding and genetics, aiming to develop next-generation hybrids that adapt to environmental changes and meet market demands.

### Literature Search and Selection Approach

This article is a narrative review informed by a structured literature search. The review aimed to synthesise evidence on conventional and contemporary approaches used in *Eucalyptus* hybrid breeding, with particular emphasis on controlled pollination, selection, genetic and genomic approaches, and emerging precision-breeding technologies. Because the objective was to integrate evidence across breeding methods, genetic resources, reproductive biology, genomics, and molecular breeding rather than to estimate a single treatment effect, a narrative review approach was considered appropriate.

The literature was assembled using major scientific databases, including Web of Science, Scopus, PubMed, and Google Scholar. Additional relevant publications were identified by examining the reference lists of key articles and authoritative reviews. Searches were conducted using combinations of keywords related to *Eucalyptus* breeding and hybridisation. The principal search terms included *Eucalyptus* AND breeding, *Eucalyptus* AND hybridization/hybridisation, *Eucalyptus* AND controlled pollination, *Eucalyptus* AND reproductive biology, *Eucalyptus* AND seed production, *Eucalyptus* AND genetic diversity, *Eucalyptus* AND QTL, *Eucalyptus* AND GWAS, *Eucalyptus* AND genomic selection, *Eucalyptus* AND marker-assisted selection, *Eucalyptus* AND genome editing, and *Eucalyptus* AND CRISPR. Additional searches combined species names, breeding methods, and economically important traits such as growth, wood properties, disease resistance, drought tolerance, and stress adaptation.

The literature search focused primarily on publications from 1990 to 2026, reflecting the rapid development of molecular and genomic technologies during this period. Earlier publications were retained when they represented foundational studies or provided important evidence on *Eucalyptus* reproductive biology, hybridisation, controlled pollination, genetic resources, or the development of breeding methodologies. The search was not restricted exclusively to *Eucalyptus* when the broader methodological literature was required to explain concepts or technologies; however, studies from other crops or tree species were not treated as direct evidence of achievements in *Eucalyptus*. Where claims concerned specific *Eucalyptus* breeding applications, preference was given to primary studies conducted in *Eucalyptus* and to authoritative *Eucalyptus*-specific reviews.

The literature was selected based on its relevance to the objectives of the review, scientific credibility, and contribution to understanding *Eucalyptus* hybrid breeding or associated technologies. Primary research articles, relevant review articles, authoritative books or book chapters, and selected theses or dissertations were considered when they provided substantive information not readily available from peer-reviewed journal articles. Publications were excluded when they were unrelated to the review objectives, lacked sufficient methodological or detailed information, or were used only to support claims for which more direct *Eucalyptus*-specific evidence was available. Particular attention was given to ensuring that citations used for *Eucalyptus*-specific claims directly supported the statements for which they were cited.

The retrieved literature was organised thematically into conventional hybridisation and controlled pollination; reproductive manipulation; genetic resources and breeding populations; molecular markers and genetic mapping; genomic selection and genome-wide association studies; and emerging genome-editing and precision-breeding approaches. Evidence was synthesised qualitatively to compare the development, applications, advantages, limitations, and future prospects of these approaches in *Eucalyptus* breeding.

## 2. Breeding Approaches and Genetic Resources of *Eucalyptus*

*Eucalyptus* genetic resources are indispensable for breeding and for meeting the growing global demand for timber, pulp, paper, and bioenergy. Key traits of economic importance in breeding programs are growth rate, wood quality, disease resistance, and environmental adaptation. Genetic variation for these traits can be harnessed through the systematic introduction of germplasm and the development of new breeding populations employing conventional and molecular breeding tools. This section synthesises approaches in the genetic enhancement of *Eucalyptus*, including germplasm introduction such as landraces and development of recombinant inbred lines (RILs), near-isogenic lines (NILs), mutant selections, and synthetic varieties, based on global research.

## 3. Germplasm Resources

### Introductions

The introduction of new *Eucalyptus* germplasm has played a key role in increasing genetic diversity and expanding the breeding base for a range of desirable traits. For instance, South Africa has prioritized the introduction of diverse genetic material, particularly in light of climatic variability and pest pressures. De Souza [13] reported that the introduction of Australian germplasm enhanced the development of drought-tolerant and *Gonipterus* weevil-resistant breeds in South Africa. The weevil is a major leaf-damaging pest in the region. Other breeding efforts have focused on improving disease resistance, such as *Chrysoporthe austroafricana* (the causal agent of stem canker), *Ceratocystis* Wilt (*Ceratocystis fimbriata* complex), and myrtle rust (*Austropuccinia psidii*) [13,14,15]. Earlier reports emphasised the importance of introducing germplasm to improve plantation productivity. Germplasm introductions have improved disease and pest resilience and have exploited the genetic bases for the development of hybrids adapted to diverse ecological conditions [16].

Genetic advances in the Brazilian forestry sector are documented through the importation of germplasm mainly from Australia, enabling breeders to develop hybrids that combine excellent growth with local adaptation. Hybrids such as *E. grandis* × *E. urophylla* are of special interest. *E. urophylla* has shown better resistance to fungal pathogens such as *Ceratocystis fimbriata*, as well as the maintenance of high wood quality and adaptability to the tropical conditions of Brazil [17].

The major *Eucalyptus* breeding aim in the USA is the development of new varieties for renewable energy applications. In the USA, *E. grandis*, *E. nitens*, and *E. smithii* have been valued for their high growth rates, biomass production, and wood density, traits important for biofuel production. The southeastern United States has had multiple introductions of these species for large-scale *Eucalyptus* plantations due to the increasing demand for biomass production [13,17]. *Eucalyptus* gene pool has been broadened in China through imports of hybrids such as *E. urophylla* × *E. grandis* from Brazil and Australia. These hybrids are reported to be important contributors to plantation productivity in southern China, where commercial forestry and bioenergy production are expanding [18].

## 4. Genetic Resources and Structured Mapping Populations in *Eucalyptus*

### 4.1. Genetic Resources and Mapping Populations Adapted to Eucalyptus Breeding

The genetic resources used in *Eucalyptus* breeding differ in important respects from those commonly employed in major crop breeding. Although structured populations such as landraces, recombinant inbred lines (RILs), and near-isogenic lines (NILs) are widely used in many crop species, they should not be assumed to represent established or routinely deployed breeding resources in *Eucalyptus*. The biology of eucalypts, including their predominantly outcrossing mating system, long breeding generation time, high heterozygosity, and substantial inbreeding depression, makes the development and maintenance of highly homozygous populations considerably more challenging than in many major food or feed crops. Controlled self-pollination can be achieved in some *Eucalyptus* species, but reduced seed production and subsequent fitness declines have been reported following selfing [19,20,21].

Consequently, *Eucalyptus* genetic studies have more commonly relied on controlled full-sib families, interspecific hybrid pedigrees, open-pollinated families, and established breeding populations for genetic dissection and trait mapping. For example, quantitative trait loci (QTL) associated with wood density, pulp yield, microfibril angle, fibre length, and cellulose content have been identified using full-sib pedigrees of *Eucalyptus* globulus [22]. Similarly, an interspecific *E. urophylla* × *E. grandis* full-sib pedigree was used to investigate the genetic architecture of wood properties, with numerous QTL detected for chemical, physical, mechanical, anatomical, and technological wood traits [23]. These studies demonstrate that biparental and full-sib pedigrees provide effective experimental populations for dissecting quantitative traits in *Eucalyptus* without requiring the development of highly homozygous RIL populations.

More recent studies have further expanded the use of structured *Eucalyptus* populations for genetic analysis. Full-sib populations derived from interspecific crosses have been used to construct linkage maps and identify QTL for growth, wood density, and other economically important traits [24]. In addition, open-pollinated families derived from mapping populations have been used to validate QTL associated with disease susceptibility and physiological disorders, demonstrating that alternative family structures can complement conventional biparental mapping approaches [25]. At the breeding-population level, genome-wide association studies and regional heritability mapping have also been applied to operational *Eucalyptus* breeding populations to identify genomic regions associated with growth, wood, and disease-resistance traits [9].

These approaches are more consistent with the reproductive biology and breeding history of *Eucalyptus* than direct transfer of population structures developed primarily for annual self-pollinating crops. In particular, the development of RILs through repeated selfing is constrained by the preferential outcrossing behaviour and inbreeding depression characteristic of many eucalypts. Studies of *E. urophylla* and *E. grandis* have demonstrated slower pollen-tube growth and reduced seed yield following self-pollination compared with cross-pollination, supporting the preferential outcrossing nature of these species [19]. Long-term studies in *E. globulus* and *E. regnans* have likewise demonstrated substantial fitness reductions associated with inbreeding [20,21]. Therefore, while RILs and NILs remain conceptually useful population structures for genetic dissection, they should not be presented as routine or established genetic resources in *Eucalyptus* breeding.

For *Eucalyptus*, future genetic dissection is therefore more appropriately supported by diverse full-sib and half-sib pedigrees, controlled interspecific hybrid populations, open-pollinated families, clonal breeding populations, and large operational breeding populations genotyped with high-density molecular markers. These population structures can be integrated with QTL mapping, genome-wide association studies, genomic selection, and other genomics-assisted approaches to exploit the high levels of genetic diversity present in eucalypt breeding populations while accounting for their predominantly outcrossing reproductive biology.

### 4.2. Interspecific Hybrids and Improved Hybrid Genotypes

Interspecific hybridisation is an important component of *Eucalyptus* breeding because it enables breeders to combine complementary characteristics from different species within a single breeding population or genotype. The economic traits associated with selected *Eucalyptus* hybrids and genotypes reported in the literature are summarised in Table 1. Interspecific combinations have been investigated and deployed to combine attributes such as rapid growth, wood quality, disease resistance, environmental adaptation, and biomass production. Among the most widely studied combinations is *Eucalyptus grandis* × *Eucalyptus urophylla*, which has been extensively used in tropical plantation forestry because of the complementary characteristics contributed by the two parental species, including growth potential and adaptation [15,26].

The performance of an interspecific hybrid depends on the parental genotypes, direction of the cross, environmental conditions, and genetic interactions between the parental genomes [27]. Consequently, hybridisation alone does not guarantee superior performance, and successful hybrid-breeding programmes require appropriate parent selection followed by controlled crossing, progeny evaluation, and selection of superior individuals or clones. In *Eucalyptus*, controlled hybridisation can also be constrained by flowering synchrony, pollen availability, reproductive compatibility, and variable seed set [28]. These reproductive factors make the integration of parent selection, pollen management, controlled pollination, and subsequent phenotypic evaluation important components of hybrid development.

Different interspecific combinations have been investigated for specific breeding objectives. Hybrids involving *E. grandis* and *E. urophylla* have received considerable attention for growth, biomass production, wood properties, and adaptation to tropical production environments [15]. Other interspecific combinations have been explored to combine growth with adaptation to particular environmental conditions or resistance to biotic and abiotic stresses. For example, *E. urophylla* × *E. pellita* has been investigated in tropical breeding programmes for characteristics including growth and disease resistance, while combinations involving *E. grandis* and *E. nitens* have been used to introduce adaptation to cooler environments [29,30]. The suitability of a particular hybrid combination therefore depends on the target production environment and the traits prioritised by the breeding programme.

**Table 1:** Selected interspecific *Eucalyptus* hybrids, breeding objective and reported deployment.

Hybrid Combination	Parental Species	Target Environment	Reported Trait Advantage	Level of Deployment/Application	References
*E. grandis* × *E. urophylla*	*E. grandis* + *E. urophylla*	Tropical and subtropical plantation environments	High growth, biomass production, wood productivity and adaptation	Widely deployed/operationally important hybrid combination	[15]
*E. grandis* × *E. nitens*	*E. grandis* + *E. nitens*	Cooler and frost-prone environments	Growth, improved form and adaptation to cooler conditions	Breeding and operational deployment in suitable environments	[29,31]
*E. urophylla* × *E. pellita*	*E. urophylla* + *E. pellita*	Warm and humid tropical environments	Growth, disease resistance and adaptation	Breeding programmes/selected deployment	[30]
*E. globulus* × *E. nitens*	*E. globulus* + *E. nitens*	Temperate environments	Growth, adaptation and wood/fibre characteristics	Breeding and field evaluation	[5]
*E. grandis* × *E. camaldulensis*	*E. grandis* + *E. camaldulensis*	Warm environments with periodic water limitation	Growth and adaptation-related traits	Breeding and evaluation	[5]
*E. globulus* × *E. viminalis*	*E. globulus* + *E. viminalis*	Temperate environments	Growth and wood-density-related traits	Experimental/breeding evaluation	[29]
*E. grandis* × *E. smithii*	*E. grandis* + *E. smithii*	Higher-altitude or cooler environments	Growth and wood-property variation	Breeding/experimental evaluation	[28,31]
*E. tereticornis* × *E. urophylla*	*E. tereticornis* + *E. urophylla*	Warm environments	Growth and adaptation-related traits	Breeding/experimental evaluation	[32]

Hybrid development has progressively incorporated molecular and genomic approaches alongside conventional phenotypic selection. Molecular markers can assist in characterising parental diversity, verifying hybrid identity, and understanding genetic relationships among breeding materials, while quantitative trait locus mapping, genome-wide association studies, and genomic selection provide additional approaches for dissecting and predicting complex traits. These technologies do not replace controlled hybridisation but can improve the efficiency with which parents and hybrid progeny are characterised and selected. Similarly, clonal propagation allows promising hybrid genotypes to be replicated and evaluated across environments before large-scale deployment.

It is important to distinguish interspecific hybrids from synthetic varieties. An interspecific hybrid is the product of a cross between genetically distinct parental individuals belonging to different species, whereas a synthetic variety represents a different breeding strategy involving the maintenance and intermating of multiple selected parental genotypes or lines. The two concepts should therefore not be treated as synonymous. In this review, the discussion is restricted primarily to interspecific hybridisation and the selection and deployment of improved hybrid genotypes because these approaches are directly relevant to *Eucalyptus* hybrid breeding.

The development of superior *Eucalyptus* hybrids is consequently best viewed as a sequential breeding process rather than simply the production of a cross. Selection of genetically complementary parents is followed by controlled pollination and hybrid seed production, verification and evaluation of progeny, selection of superior individuals, clonal propagation, and multi-environment testing. Integrating phenotypic, reproductive, molecular, and genomic information across these stages can improve the efficiency of hybrid development and increase the likelihood that selected genotypes combine productivity, quality, adaptation, and resistance to important biotic and abiotic stresses.

### 4.3. Eucalyptus Genetic Resource Collections and Genomic Databases

The long-term success of *Eucalyptus* breeding depends on maintaining and effectively utilising genetic diversity to develop improved planting material for changing environmental and production conditions [33]. Genetic resource collections and genomic databases provide complementary infrastructure for the conservation, characterization, and utilisation of *Eucalyptus* genetic diversity. Physical collections preserve germplasm for future use, whereas genomic databases facilitate access to genome-scale information that can support genetic analysis, trait discovery, and molecular breeding. Selected genetic resource collections and genomic databases relevant to *Eucalyptus* breeding and conservation are presented in Table 2 [34].

Ex situ conservation of *Eucalyptus* and related *Corymbia* germplasm is undertaken through established seed collections. The Australian Tree Seed Centre (ATSC), managed by CSIRO, maintains a large collection of tree seed, including extensive representation of *Eucalyptus* and *Corymbia*, and provides documented genetic material for research, conservation, and breeding applications [35]. In Brazil, the Embrapa Florestas seed bank maintains *Eucalyptus* and *Corymbia* germplasm, including approximately 3000 seed samples representing 68 species and material from different provenances and progenies [36]. Such collections provide an important source of genetic variation that can be conserved outside natural populations and subsequently accessed for research and breeding.

The conservation of germplasm is particularly important for *Eucalyptus* because commercial breeding can result in the intensive use of a relatively limited number of elite parents and clones. Conserved genetic resources can therefore provide access to genetic variation that may be underrepresented in advanced breeding populations. However, the value of a gene bank depends not only on the preservation of material but also on accurate documentation, characterization, and accessibility of the conserved germplasm. These factors facilitate the identification and incorporation of useful genetic variation into breeding populations.

Genomic databases complement physical germplasm collections by providing access to sequence, genetic, transcriptomic, and other molecular information. The EucGenIE resource provides genomic and transcriptomic information for *Eucalyptus grandis* and has supported genome annotation, gene-expression analysis, and functional genomic studies [37,38]. EUCANEXT provides additional genomic and transcriptomic resources for *Eucalyptus*, including expressed-sequence-tag information, thereby supporting comparative and functional genomic research [39,40]. These resources have contributed to the development of genomic information required for the characterization of genes, genetic variation, and biological processes relevant to *Eucalyptus* improvement.

More recently, EucaMOD (*Eucalyptus* multi-omics database) has expanded the available genomic infrastructure by integrating multiple data types, including genome, comparative-genome, pan-proteome, transcriptome, epigenome, and genetic-variation information [41]. The integration of these datasets provides a broader framework for investigating gene function, genetic variation, and trait-associated molecular information across *Eucalyptus* species. Such resources can complement conventional breeding by facilitating the identification and interpretation of candidate genomic regions and molecular markers relevant to traits of breeding importance.

Together, physical genetic resource collections and genomic databases provide complementary components of *Eucalyptus* genetic-resource management. Seed collections maintain biological material that can be accessed for future breeding and research, while genomic databases facilitate the characterization and analysis of the genetic information associated with that material. Their integration with molecular breeding approaches, including marker-assisted selection, quantitative trait locus analysis, genome-wide association studies, and genomic selection, provides opportunities to improve the efficiency with which conserved genetic diversity is evaluated and incorporated into *Eucalyptus* breeding programs.

### 4.4. Comparative Genetics Between Eucalyptus and Populus

A comparison with other fast-growing forest trees, particularly Populus, provides useful context for understanding the distinctive features of *Eucalyptus* genomics-assisted breeding. Both genera have been important models for investigating the genetic regulation of growth, environmental adaptation, and wood formation, and both exhibit substantial genetic variation for traits associated with drought response, nutrient acquisition, biomass production, and secondary cell-wall development. In Populus, genomic and transcriptomic studies have contributed substantially to the identification of genes and regulatory networks involved in abiotic stress responses and wood formation, including pathways controlling cellulose, hemicellulose, and lignin biosynthesis [42]. Similar molecular networks operate in *Eucalyptus*, but their expression and contribution to phenotypic variation can differ among species, environments, and developmental stages.

Despite these similarities, *Eucalyptus* breeding has several distinctive characteristics arising from its exceptional growth rates, broad species diversity, adaptation to tropical and subtropical environments, and extensive use of interspecific hybridization. The genus also presents substantial opportunities for exploiting species-specific and interspecific genetic variation for drought tolerance, disease resistance, wood quality, and biomass productivity. Furthermore, the comparatively complex reproductive biology and the importance of controlled hybridization in commercial *Eucalyptus* breeding create a breeding context that differs from the predominantly clonal and hybrid breeding systems commonly used in Populus [42]. Consequently, genomic tools in *Eucalyptus* are particularly valuable when integrated with controlled pollination, interspecific hybridization, and field-based selection rather than being considered as independent technologies. Comparative knowledge from Populus therefore provides a useful molecular framework while highlighting the need to develop *Eucalyptus*-specific genomic resources and breeding strategies for its diverse production environments and industrial applications.

## 5. Induced Mutagenesis

Induced mutations enhance genetic variation for breeding. Spontaneous mutation occurs less frequently, requiring induced mutagenesis, selection and controlled hybridisation [43]. Induced mutagenesis has provided genetic variation for growth performance, wood quality, and environmental stress tolerance, although it is not as widely used in *Eucalyptus* breeding programs compared to hybridisation. Mutation breeding adds another source of heritable variation that can be used to improve conventional breeding methods. For example, in Brazil, Ziegler et al. [44] successfully induced mutations in *E. saligna* and *E. dunnii* using gamma irradiation, demonstrating the potential of mutagenesis as a complementary tool for *Eucalyptus* genetic improvement.

## 6. Market-Driven Applications of Eucalypt Breeding

*Eucalyptus* breeding is market-driven, transforming the breeding objectives from general productivity improvement to the development of genotypes adapted to specific industrial uses. Modern breeding programmes select trees for wood quality, resistance to biotic and abiotic stresses, and adaptability to local environments, rather than only fast growth, to maximise plantation profitability and long-term sustainability.

Each industry has different desired breeding objectives. For the pulp and paper industry, high cellulose content, desirable fibre properties, and low levels of extractives that hinder pulping are important, while for the solid wood industry, high wood density, stem straightness, mechanical strength, and fast growth are valued [45]. Consequently, interspecific hybrids are increasingly used in breeding programmes to combine complementary traits. For example, hybrids of *E. grandis* × *E.urophylla* are widely used due to their fast growth and higher resistance to diseases, pests, and environmental conditions, and are used in commercial plantations in some tropical and subtropical areas [15,46].

In addition to traditional timber production, there are new breeding goals due to increased interest in renewable energy. Bioenergy programmes are targeting genotypes with high biomass yield, good lignocellulosic composition, and efficient nutrient use. At the same time, increasing climate variability has increased the importance of drought tolerance, resource-use efficiency, and carbon sequestration potential, making *Eucalyptus* breeding an important component of climate-smart forestry [12].

Market requirements are also driven more by the need to minimise production risks from pests and diseases. The presence and spread of pathogens and insect pests have altered breeding targets, leading to the development of resilient planting material that can sustain productivity while minimising the need for chemical control measures. Resistance to major threats of diseases and insects, such as *Puccinia psidii*, *Leptocybe invasa*, and *Gonipterus* species, has therefore become an integral part of breeding programmes, especially when these biotic agents represent a significant constraint on plantation productivity [26]. Developments show that modern *Eucalyptus* breeding is becoming more market-oriented, with breeding objectives changing in response to evolving industrial demands, environmental challenges, and sustainability goals, rather than focusing solely on improvements in growth performance.

At the molecular level, these market-driven traits are controlled by interconnected regulatory networks governing secondary cell-wall formation and lignocellulose biosynthesis. In particular, the phenylpropanoid and monolignol pathways regulate lignin formation through enzymes such as phenylalanine ammonia-lyase (PAL), cinnamate 4-hydroxylase (C4H), 4-coumarate:CoA ligase (4CL), cinnamoyl-CoA reductase (CCR), cinnamyl alcohol dehydrogenase (CAD), and caffeoyl-CoA O-methyltransferase (CCoAOMT), while cellulose and hemicellulose deposition are regulated through coordinated carbohydrate biosynthetic pathways and secondary cell-wall regulatory networks. These molecular determinants provide potential targets for directional improvement of wood quality and biomass composition [47]. Genomics-assisted approaches, including QTL mapping, GWAS, transcriptome analysis, marker-assisted selection, and genomic selection, can be used to identify alleles, regulatory regions, and candidate genes associated with lignin content, cellulose accumulation, fibre characteristics, and wood density, thereby enabling these traits to be incorporated into breeding decisions [9,12]. Where candidate genes with strong functional effects are identified, genome-editing technologies such as CRISPR-Cas systems offer a potential approach for targeted validation or modification of specific genes within these pathways [48,49]. For example, modulation of genes involved in monolignol biosynthesis could potentially alter lignin quantity or composition, with implications for pulping efficiency and lignocellulosic biomass conversion. However, the application of genome editing for commercial improvement of these complex wood traits in *Eucalyptus* remains an emerging area, and the extent to which individual genes can be predictably manipulated without affecting growth, structural integrity, or other economically important traits requires further validation.

## 7. Breeding Gains in *Eucalyptus*

### 7.1. Realized Genetic Gains

Genetic improvement programmes for *Eucalyptus* plantations have greatly enhanced the productivity, adaptability, and commercial value of *Eucalyptus* plantations [12]. Breeding strategies have differed among countries, reflecting local market requirements, environmental conditions and industrial priorities. Still, all have demonstrated that sustained selection and hybridisation can generate cumulative genetic gains that improve plantation performance and support the long-term sustainability of the forestry sector. These realised benefits include higher growth rates, better tolerance to environmental stresses, improved wood quality, and greater resilience under changing climatic conditions.

### 7.2. Productivity Gains

Improved growth rate and biomass production remain the primary objectives of most *Eucalyptus* breeding programmes because they directly influence plantation profitability and wood supply [18]. The popularity of hybrids such as *E. grandis* × *E. urophylla* demonstrated the synergy of complementary traits from different species, improving growth performance while maintaining adaptability across different plantation conditions [50]. These genetic gains have translated into shorter rotation ages, increased wood production per unit area, resource-use efficiency, and enhanced economic competitiveness of commercial forestry.

### 7.3. Enhanced Resistance to Biotic and Abiotic Stresses

As climate variability and pests have become more pressing, breeding programmes have increasingly focused on resilience and productivity. Selection for drought tolerance has become more relevant in water-limited production environments, where survival and growth under moisture stress are important for the stability of the plantations [32]. In addition, the breeding of resistance to pests and diseases of economic importance has reduced production losses and favoured more sustainable plantation management by reducing dependence on chemical control methods [51]. These advances demonstrate the fundamental role of genetic improvement in increasing the adaptive capacity of *Eucalyptus* plantations to changing environmental conditions.

Salinity represents an additional abiotic constraint that is becoming increasingly relevant where *Eucalyptus* is cultivated on marginal, irrigated, coastal, or salt-affected lands [52]. Physiological studies have demonstrated substantial clonal and species-level variation in salinity responses, indicating opportunities for selection of tolerant planting material [53]. Under saline conditions, tolerant *Eucalyptus* genotypes can maintain comparatively higher photosynthetic activity, stomatal function, photochemical efficiency, pigment stability, and biomass accumulation, whereas susceptible genotypes exhibit stronger reductions in gas exchange and growth [52,53]. Screening of *Eucalyptus* clones under saline conditions has therefore identified physiological and biochemical traits that can be used as indicators of salt tolerance [32]. Recent large-scale screening of *E. camaldulensis* and *E. tereticornis* clones further demonstrated considerable variation in physiological, biochemical, and growth responses to severe salinity, supporting the use of clonal selection for salt-affected environments [32].

The physiological response of *Eucalyptus* to salinity involves both osmotic and ionic components. Salinity can restrict water uptake, alter stomatal behaviour and photosynthesis, promote oxidative stress, and disturb cellular ion homeostasis. Comparative studies among *Eucalyptus* genotypes have shown that salt tolerance is associated with differences in ionic regulation, antioxidant responses, photosynthetic maintenance, and water relations [54]. A naturally occurring *Eucalyptus* hybrid adapted to salinized floodplain conditions also demonstrated physiological characteristics associated with tolerance, including the ability to maintain lower plant water potentials and extract water under conditions of reduced soil water availability [55]. These findings indicate that physiological variation can provide a useful basis for identifying parental material for breeding programmes targeting saline environments.

Molecular studies are beginning to clarify the regulatory mechanisms underlying these phenotypic differences. Transcriptomic and gene-expression studies in *Eucalyptus* have implicated several stress-responsive regulatory systems, including abscisic acid (ABA) signalling, ethylene responses, mitogen-activated protein kinase (MAPK) cascades, antioxidant defence, carbohydrate metabolism, and transcription-factor networks [54,56]. For example, recent comparative transcriptomic analysis of contrasting *Eucalyptus* clones exposed to prolonged salinity identified coordinated activation of ABA and ethylene signalling, MAPK pathways, antioxidant responses, carbohydrate metabolism, protein-folding processes, and genes encoding dehydrins, ethylene-response factors and expansins in the more salt-tolerant clone [54]. Other studies have identified salt-responsive transcription-factor families, including FAR1/FHY3, HD-Zip and NF-YB, providing candidate regulatory components for further functional validation [54,55,56].

Molecular breeding approaches have also provided proof-of-concept for improving salinity tolerance in *Eucalyptus*. Transgenic *Eucalyptus camaldulensis* expressing an RNA-binding protein gene (McRBP) from the halophytic species Mesembryanthemum crystallinum showed improved survival, growth, photosynthetic performance, and reduced leaf salt accumulation under experimental salinity [57]. Similarly, transgenic *E. globulus* expressing the codA gene showed increased glycinebetaine accumulation and improved tolerance to salt stress [58]. These studies demonstrate the potential of molecular approaches for manipulating salt-response mechanisms; however, they remain experimental and should not be interpreted as evidence that transgenic salt-tolerant *Eucalyptus* is already an established operational breeding technology.

The available evidence indicates that salinity tolerance in *Eucalyptus* is genetically variable and physiologically complex. Current opportunities include screening diverse clones and breeding populations, characterising physiological indicators of tolerance, integrating transcriptomic and genomic information to identify candidate genes and regulatory pathways, and subsequently validating candidate loci through marker-assisted or genomic approaches. However, compared with growth and wood-property traits, molecular breeding specifically targeting saline-alkaline tolerance remains comparatively less developed. Further integration of physiological screening, controlled hybridisation, high-throughput genotyping, transcriptomics, and multi-environment field evaluation could therefore facilitate the development of *Eucalyptus* hybrids better adapted to salt-affected and other marginal environments.

### 7.4. Improvements in Wood Quality

Genetic improvement has also targeted wood properties that determine industrial performance. Modern breeding programmes are increasingly focused on the selection for improved wood density, fibre properties, and cellulose content, rather than simply selecting for maximum growth as dictated by the needs of different forest industries [45]. Improvements in these traits have resulted in better-quality raw materials for pulp and paper production and more suitable *Eucalyptus* biomass for renewable energy applications. This change reflects the increasing recognition that breeding goals must balance productivity and end-use quality to optimize economic benefits along multiple value chains [59].

The phenotypic improvement of wood density and fibre characteristics is underpinned by complex molecular networks controlling secondary cell-wall formation, including the biosynthesis and deposition of cellulose, hemicellulose, and lignin. Lignin biosynthesis is particularly important because both lignin concentration and monolignol composition influence wood structure, fibre properties, pulp yield, and the efficiency of biomass processing [60]. Regulatory transcription factors, including members of the NAC and MYB families, coordinate the expression of downstream genes involved in secondary cell-wall biosynthesis, while enzymes of the phenylpropanoid and monolignol pathways, including PAL, 4CL, CCR, CAD, C4H, and CCoAOMT, contribute directly to lignin formation [47]. Cutting-edge breeding technologies can exploit this molecular architecture at different levels. QTL mapping and GWAS can identify genomic regions associated with wood density, fibre traits, cellulose and lignin characteristics, whereas transcriptomic and other omics approaches can help identify genes and regulatory networks underlying variation in these traits. Marker-assisted and genomic selection can subsequently incorporate favourable genetic variation into breeding populations, particularly for traits controlled by many loci. In addition, CRISPR-Cas genome editing provides a prospective strategy for functionally validating candidate genes and, where appropriate, directionally modifying specific components of lignin or secondary cell-wall pathways. Such targeted interventions could potentially reduce undesirable lignin characteristics or modify lignin composition while maintaining cellulose deposition and tree growth [49]. Nevertheless, because wood properties are highly quantitative and influenced by genotype, environment, developmental stage, and interactions among metabolic pathways, molecular manipulation should be coupled with phenotypic and field-based evaluation to ensure that improvements in processing characteristics do not compromise tree growth, wood strength, or plantation performance.

### 7.5. Sustainable Approaches

In addition to increasing plantation productivity, genetic improvement has wider economic and environmental benefits. Improved planting material has been widely adopted, supporting the expansion of commercial forestry and the competitiveness of the forest sector in several African countries [61,62]. Additionally, breeding programmes are increasingly incorporating traits related to water-use efficiency, climate adaptation and long-term plantation resilience, reflecting the shift to climate-smart forestry. Therefore, the realised genetic gains are now assessed not only based on yield improvement but also on their contribution to sustainable forest management, resource-use efficiency and the long-term resilience of plantation systems.

In general, breeding programmes globally demonstrate the benefits of continuous genetic improvement, which accrue over time as productivity, adaptation, wood quality, and sustainability are integrated. The results show the evolution of *Eucalyptus* breeding from a science primarily concerned with yield improvement to one that addresses the broader problems of modern plantation forestry.

## 8. Breeding for Climate-Smart Forestry

### 8.1. Below-Ground Phenotypic Traits

Below-ground traits are an important but comparatively less explored component of climate-smart *Eucalyptus* breeding. Root system architecture, including rooting depth, root length, root diameter, lateral root development, root biomass, and root distribution, directly influences water and nutrient acquisition, drought adaptation, tree stability, and the capacity of forest systems to retain carbon below ground. Root traits also influence carbon allocation to soils through root turnover, rhizodeposition, and interactions with soil microbial communities [63]. Consequently, incorporating below-ground traits into breeding objectives could complement improvements in above-ground biomass and wood production and contribute to more resilient plantation systems under increasing climatic variability.

The molecular regulation of root development involves complex genetic and physiological networks controlling root initiation, elongation, branching, hormonal signalling, and responses to water and nutrient availability. Genomics-assisted approaches, including QTL mapping, GWAS, transcriptomics, and genomic selection, provide opportunities to identify genomic regions, candidate genes, and regulatory networks associated with root architecture, drought responsiveness, water-use efficiency, and below-ground biomass [12]. High-throughput root phenotyping and imaging can further facilitate the measurement of root-system traits and enable their integration with genomic information. Where functional candidate genes are identified, genome-editing technologies such as CRISPR-Cas systems may provide a prospective approach for validating their roles in root development and stress adaptation. However, root traits are strongly influenced by soil properties, environmental conditions, tree age, and interactions with the soil microbiome, making their phenotyping and genetic improvement particularly challenging. Future *Eucalyptus* breeding programmes should therefore integrate below-ground phenotyping with genomic selection and multi-environment evaluation to identify genotypes that combine productive above-ground growth with efficient rooting, enhanced environmental resilience, and greater potential for long-term below-ground carbon storage.

### 8.2. Genomic-Assisted Breeding

**Figure 1:**
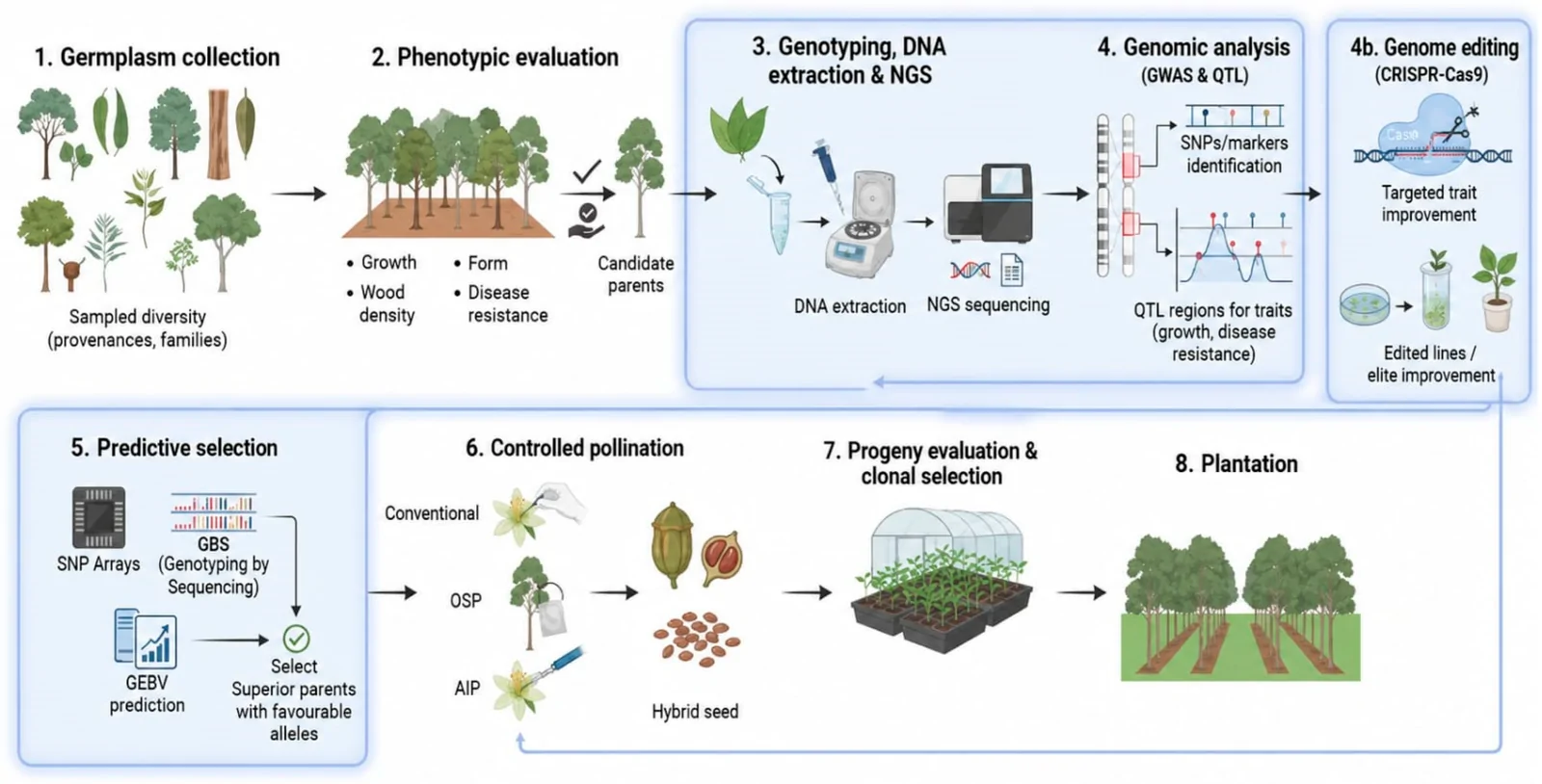
Schematic representation of an integrated traditional and genomic-assisted breeding strategy in *Eucalyptus* showing the sequential steps from germplasm collection and phenotypic evaluation to genomic analysis, predictive selection, controlled pollination, progeny evaluation, clonal selection and commercial plantation establishment. Notes: DNA, deoxyribonucleic acid; NGS, next-generation sequencing; GWAS, genome-wide association study; QTL, quantitative trait locus; SNP, single nucleotide polymorphism; GBS, genotyping-by-sequencing; GEBV, genomic estimated breeding value; OSP, One-Stop Pollination; AIP, Artificially Induced Protogyny.

Integrating phenotypic selection with genomic-assisted breeding is illustrated in Figure 1. The incorporation of genomic information into breeding programmes has transformed the improvement of *Eucalyptus* by providing breeders with the ability to identify desirable alleles, predict breeding values, and enhance the accuracy of selection for complex traits. The advances in high-throughput genotyping, genome-wide marker discovery, and sequencing technologies have greatly improved our knowledge of the genetic architecture controlling growth, wood quality, disease resistance, and abiotic stress tolerance [64].

#### 8.2.1. Genotype Selection Using Biochemical Markers

Biochemical markers, such as enzymes, proteins, and other metabolites, have been used in *Eucalyptus* breeding to evaluate genetic variation and aid in selecting desirable traits. They are detected using laboratory methods such as electrophoresis and chromatography, and are an indirect selection criterion, but an efficient way to assess genetic variation among individuals within a breeding population [12].

A commonly used technique to identify genetic diversity within and between species of *Eucalyptus* is the analysis of enzyme variants, or isozymes. These have proved useful in conservation programs and in the selection of individuals exhibiting resistance to pests such as the *Eucalyptus* gall wasp [65]. In a similar vein, differences in protein expression are linked to traits such as drought tolerance and wood composition for early-stage selection without requiring mature phenotypic traits.

Biochemical markers are less precise than DNA-based markers but have practical advantages, especially in the absence or costliness of molecular tools. But their sensitivity to environmental conditions that can affect metabolite and enzyme levels poses a challenge. Nevertheless, as analytical techniques improve, biochemical markers are expected to become more useful, especially in hybrid breeding, where rapid screening is advantageous, as a supportive component in the early-stage selection and resource conservation in *Eucalyptus* breeding [66].

#### 8.2.2. Genotyping

The efficiency of *Eucalyptus* breeding has been significantly increased by high-throughput genotyping, allowing breeders to characterize genetic variation at the genome level and use this information in the selection process [67]. Genotyping allows the detection of favourable alleles before the full manifestation of traits, unlike conventional breeding, thus increasing the accuracy of selection and reducing the time to identify superior breeding material.

The increasing availability of single nucleotide polymorphism (SNP) markers, simple sequence repeats (SSRs), and genotyping-by-sequencing (GBS) platforms has broadened the ability to evaluate large breeding populations while facilitating genome-wide association studies and trait mapping [68]. These advances have enhanced the identification of genomic regions associated with economically important traits such as growth, wood quality, disease resistance, and adaptation to the environment. Genotyping has thus become a key element of contemporary *Eucalyptus* breeding as it provides the genomic basis for downstream applications such as marker-assisted selection, genomic selection and predictive breeding.

#### 8.2.3. Genomic Selection and Genomic Prediction

Genomic selection (GS) and genomic prediction (GP) represent important developments in the application of genomics to *Eucalyptus* breeding, particularly for complex quantitative traits controlled by many loci with relatively small effects. In contrast to marker-assisted selection (MAS), which generally focuses on markers linked to specific genomic regions or loci of relatively large effect, genomic selection uses genome-wide molecular markers to estimate the breeding value of individuals. This approach is particularly relevant to perennial tree breeding, where phenotyping mature traits can require substantial time, land, and financial resources [12].

A genomic selection programme generally consists of a phenotype and genotype training population used to develop a statistical prediction model. Genome-wide marker data are combined with phenotypic and, where available, pedigree information to estimate the relationships between marker variation and breeding performance [9]. The resulting model can then be used to predict genomic estimated breeding values (GEBVs) for genotyped selection candidates. Individuals with favourable predicted breeding values can be prioritised for further evaluation or breeding before complete phenotypic information is available. The accuracy of genomic prediction depends on factors including the size and genetic composition of the training population, marker density, trait heritability, relatedness between the training and prediction populations, and the statistical model used [12].

The potential value of genomic selection is particularly high for *Eucalyptus* because many breeding objectives are complex and are influenced by numerous genes and environmental conditions. Traits such as diameter growth, height, wood density, fibre properties, disease resistance, and adaptation are generally quantitative and may be difficult to improve efficiently using a small number of markers associated with individual quantitative trait loci. Genome-wide prediction can capture the cumulative contribution of many loci, including loci with small effects that may not be individually detectable through conventional QTL mapping. Consequently, genomic selection provides a complementary strategy to QTL-based MAS for complex traits [9].

Genomic selection can also contribute to shortening the breeding cycle of *Eucalyptus*. Conventional selection may require several years of field evaluation before the performance of individuals for mature traits can be assessed reliably [12]. Genomic prediction provides an opportunity to select individuals at an earlier developmental stage when reliable prediction models are available, thereby reducing the number of candidates that need to be retained for long-term field testing. This does not eliminate the need for phenotypic evaluation because prediction models require high-quality phenotypic training data and their accuracy must be validated in relevant genetic and environmental populations. Instead, genomic selection can be used to prioritise individuals for subsequent controlled crosses, clonal testing, and multi-environment evaluation.

Several studies have demonstrated the potential of genomic prediction in *Eucalyptus* breeding populations for growth and wood-related traits [6,9,12,69,70,71]. The application of dense SNP genotyping has enabled the estimation of genomic breeding values and the evaluation of prediction accuracy for quantitative traits, providing evidence that genome-wide information can contribute to selection decisions in operational tree-improvement contexts [67,68]. Prediction performance, however, varies among traits, populations, environments, and validation strategies. Models trained in one population or environment may lose accuracy when applied to genetically distant populations or different environments, emphasising the importance of appropriate training-population design and periodic model updating.

An important opportunity is the integration of genomic selection with controlled hybrid breeding. Genomic information can first contribute to the characterization and selection of complementary parents, while controlled pollination is used to generate targeted hybrid combinations. Progeny can subsequently be genotyped and phenotyped to expand or update the training population, and genomic prediction can be used to prioritise superior individuals for clonal propagation and field testing. In this way, genomic selection does not replace conventional hybridisation but strengthens decision-making at several stages of the breeding pipeline.

The integration of genomic prediction with phenotypic information may also improve selection for traits that are expensive, difficult, or time-consuming to measure [69]. Multi-trait genomic prediction can potentially exploit genetic correlations among traits, while models incorporating environmental information may improve prediction across contrasting production environments. Such approaches are particularly relevant where breeders must simultaneously consider productivity, wood quality, disease resistance, and adaptation.

Despite its potential, genomic selection requires substantial investment in genotyping, accurate phenotyping, statistical modelling, and long-term management of training populations. Prediction accuracy can decline when marker effects, genetic relationships, or environmental conditions differ between the training and target populations. Furthermore, genomic prediction should not be interpreted as a substitute for field validation, particularly for long-lived perennial trees where genotype × environment interactions can influence commercial performance.

Genomic selection provides a potentially powerful complement to conventional phenotypic selection, MAS, and QTL-based approaches in *Eucalyptus* breeding [69,70,71]. Its greatest value is likely to occur when genome-wide prediction is integrated with carefully designed breeding populations, controlled hybridisation, clonal testing, and multi-environment evaluation. Such integration can improve the efficiency of selecting superior parents and progeny, reduce the number of individuals requiring extensive field testing, and potentially shorten the breeding cycle for complex traits.

#### 8.2.4. High-Throughput Molecular Verification and Early Generation Selection of Hybrids

The integration of molecular markers with controlled hybridization becomes particularly valuable when large numbers of progeny are generated through techniques such as AIP, OSP, or conventional controlled pollination. In commercial breeding programmes, the production of large progeny populations can result in substantial numbers of seedlings that require verification of parentage and genetic identity before they enter subsequent field evaluation [28]. SNP-based genotyping provides a high-throughput approach for addressing this challenge by allowing large numbers of seedlings to be genotyped simultaneously and compared with the known parental genotypes. Genome-wide SNP profiles can be used to confirm whether progeny possess genetic contributions from both intended parents, identify self or unintended pollen contamination, and detect individuals with inconsistent parentage. This provides an important quality-control step following controlled hybridization, particularly where large-scale seed production makes morphological verification of hybrid status impractical.

Beyond hybrid-purity verification, SNP genotyping can also support early selection for favourable genetic variation. Once alleles or genomic regions associated with economically important traits such as growth, wood quality, disease resistance, drought adaptation, or other breeding objectives have been identified through QTL mapping, GWAS, or genomic studies, SNP markers can be used to screen large numbers of young progeny before expensive and time-consuming field evaluation. Individuals carrying favourable alleles or genomic profiles can therefore be prioritised for nursery or field testing, while genetically unsuitable individuals can be removed at an early stage [9,12]. In this way, molecular screening does not replace phenotypic selection but reduces the number of individuals that need to be evaluated intensively and improves the efficiency of downstream breeding operations.

The commercial value of this integration is particularly evident when reproductive technologies are used to generate large progeny populations. AIP or OSP can increase the efficiency of controlled seed production, while SNP-based genotyping provides a subsequent mechanism for verifying the genetic identity of the resulting progeny and prioritising individuals with desirable genetic profiles. The combined workflow can therefore be conceptualised as: controlled hybridization → large progeny production → SNP-based parentage and hybrid verification → favourable allele/genomic screening → early selection → field evaluation of selected individuals. Such integration has the potential to shorten breeding cycles, reduce field-testing costs, and improve the efficiency with which superior hybrid combinations are identified. However, the reliability of favourable-allele screening depends on the strength and validation of marker–trait associations, and genomic predictions or marker effects should be validated across relevant genetic backgrounds and environments before being used for large-scale commercial selection.

#### 8.2.5. Marker-Assisted Selection

Marker-assisted selection (MAS) represented one of the earliest successful applications of molecular genetics in *Eucalyptus* breeding by allowing breeders to incorporate DNA marker information into conventional selection programmes. The greatest contribution of MAS has been its ability to improve selection efficiency for traits controlled by major genes, particularly where phenotypic evaluation is expensive, time-consuming, or strongly influenced by environmental variation [72].

Despite these advantages, the application of MAS in *Eucalyptus* has been constrained because many commercially important traits, including growth, wood quality, and drought tolerance, are quantitatively inherited and controlled by numerous genes with relatively small individual effects [73]. As a result, MAS is increasingly viewed as a complementary breeding tool rather than a standalone strategy. Nevertheless, it continues to play an important role in validating favourable alleles and supporting breeding programmes focused on disease resistance and other traits with relatively simple genetic architecture. Recent advances in genomic selection have extended the principles of MAS by including information from thousands of genome-wide markers, thereby improving prediction accuracy for complex traits.

#### 8.2.6. Quantitative Trait Loci (QTL) Analysis

Quantitative trait locus (QTL) analysis has been an important tool for understanding the genetic architecture of complex traits in *Eucalyptus*. QTL studies have identified genomic regions associated with economically important characteristics, including growth performance, wood density, fibre properties, and resistance to pests and diseases [9].

The major scientific contribution of QTL analysis has been the identification of genomic regions associated with phenotypic variation, providing insights into trait inheritance and supporting the development of molecular markers for breeding programmes [74]. However, many QTLs have relatively small effects, and their expression may vary among genetic backgrounds and environments, limiting their direct application in operational breeding [69]. Consequently, QTL mapping is increasingly integrated with genome-wide approaches, including genome-wide association studies and genomic selection, which use information from multiple loci to improve the prediction of complex traits [75].

An important next step after QTL identification is the identification and functional validation of candidate genes located within or near trait-associated genomic regions. QTL mapping alone establishes statistical association between genomic regions and phenotypic variation but does not necessarily demonstrate that a particular gene is causally responsible for the observed phenotype [9]. Functional validation is therefore required to determine whether candidate genes influence the biological processes underlying the target trait.

Several molecular interaction and regulatory assays can contribute to this validation process. Yeast Two-Hybrid (Y2H) analysis can be used to investigate protein–protein interactions and determine whether a candidate protein physically interacts with other components of a stress-response, developmental, or metabolic pathway [76]. Bimolecular Fluorescence Complementation (BiFC) provides complementary evidence by visualising protein–protein interactions in living cells and can provide information about their subcellular localisation [77]. Co-Immunoprecipitation (Co-IP) can further validate protein associations under more native cellular conditions, particularly when candidate proteins are suspected to participate in multi-protein complexes [78].

For candidate transcription factors, Electrophoretic Mobility Shift Assay (EMSA) provides a means of testing whether a protein binds directly to a specific DNA sequence or promoter element [79]. This is particularly relevant for *Eucalyptus* candidate genes associated with abiotic-stress responses, where transcriptomic and genomic studies may identify transcription factors but do not by themselves establish direct regulation of downstream genes. Combining EMSA with promoter analysis and gene-expression studies can therefore help establish regulatory relationships between candidate transcription factors and stress-responsive target genes [76,79].

These approaches should be regarded as complementary rather than interchangeable. Y2H, BiFC, and Co-IP primarily provide evidence concerning protein–protein interactions, whereas EMSA addresses direct protein–DNA binding [76,77,78,79]. A stronger functional-validation framework can therefore combine multiple approaches with gene-expression analysis, promoter assays, heterologous expression, gene overexpression or silencing, and, where technically and ethically appropriate, genome editing. In *Eucalyptus*, such integrated validation could be particularly valuable for candidate genes associated with growth, wood formation, drought, cold, salinity, and disease resistance.

However, the availability of a candidate gene does not automatically justify its immediate deployment as a breeding marker. Functional validation should ideally establish the biological relevance of the candidate, confirm the direction and context of its effect, and evaluate whether the association is reproducible across genetic backgrounds and environments. For forest trees, this is particularly important because long generation times, extensive genotype-by-environment interactions, and the complexity of quantitative traits can make translation from molecular discovery to operational breeding challenging.

QTL analysis provides an important entry point for candidate-gene discovery, while molecular interaction and functional assays can help bridge the gap between statistical association and biological mechanism. Integrating QTL/GWAS results with Y2H, BiFC, Co-IP, EMSA, transcriptomics, gene-expression analysis, and functional genetics offers a more complete framework for validating candidate genes before their incorporation into marker-assisted or genomic breeding strategies in *Eucalyptus*.

#### 8.2.7. Next-Generation Sequencing and Multi-Omics Integration

Next-generation sequencing (NGS) has substantially expanded the genomic resources available for *Eucalyptus* and has become an important component of contemporary breeding research. High-throughput sequencing can generate genome-wide information for marker discovery, genetic diversity assessment, genome-wide association studies, transcriptome profiling, comparative genomics, and the development of genomic prediction models [70,71]. These resources have improved the resolution with which genetic variation can be investigated and have contributed to the identification of genes and genomic regions associated with growth, wood formation, disease resistance, and environmental adaptation.

However, genomic information alone does not fully explain the biological processes responsible for complex quantitative traits. Traits such as growth rate, wood density, lignin content, fibre characteristics, secondary metabolite composition, and stress tolerance arise from interactions among multiple genes, regulatory processes, proteins, metabolic pathways, and environmental conditions [70]. Consequently, integration of multiple molecular data types can provide a more complete understanding of the mechanisms connecting genetic variation with phenotype. Multi-omics approaches are particularly relevant to *Eucalyptus* because many economically important traits are quantitatively inherited and are influenced by genotype-by-environment interactions [80].

Transcriptomics provides a direct means of examining how candidate genes respond across tissues, developmental stages, genotypes, and environmental conditions [80]. RNA sequencing can identify differentially expressed genes, alternative transcripts, co-expressed gene networks, and regulatory pathways associated with variation in target traits. In *Eucalyptus*, transcriptomic studies have been extensively applied to wood formation, abiotic stress responses, disease resistance, and secondary metabolism [81]. Transcript information can therefore be used to prioritise candidate genes identified through QTL mapping or genome-wide association studies, particularly when genetic variants are associated with differences in gene expression [81].

Proteomics provides a complementary layer of information because transcript abundance does not necessarily correspond directly to protein abundance or enzyme activity [82]. Post-transcriptional and post-translational regulation can alter the amount, stability, localisation, or activity of proteins involved in developmental and metabolic processes [82]. Integrating transcriptomic and proteomic information can therefore help determine whether changes in gene expression are reflected at the protein level [81,82]. This is particularly relevant for wood formation and secondary metabolism, where enzymes involved in cellulose, hemicellulose, lignin, terpenoid, and phenylpropanoid pathways ultimately determine the biochemical processes that contribute to wood and defence-related phenotypes.

Metabolomics provides a further downstream layer by measuring changes in small molecules that represent the biochemical consequences of genetic and environmental variation. Among available analytical platforms, gas chromatography-mass spectrometry (GC-MS) is particularly useful for profiling volatile and derivatisable primary and secondary metabolites. In *Eucalyptus*, metabolomic profiling can be used to investigate changes in compounds associated with carbon metabolism, phenylpropanoid pathways, terpenoid metabolism, defence responses, and other processes that contribute to wood chemistry and adaptation. Importantly, metabolite profiles can sometimes provide a closer biochemical representation of the phenotype than transcript abundance alone.

Evidence from *Eucalyptus* demonstrates the value of combining these approaches. An integrated study of *Eucalyptus grandis* bark examined changes in gene expression, proteins, and metabolites using transcript analysis, proteomics, and GC-MS metabolomics under contrasting seasonal conditions [83]. The combined analysis showed that seasonal variation was associated with coordinated changes at transcript, protein, and metabolite levels, illustrating how multi-layered data can be used to investigate metabolic regulation in a forest tree [83]. Similarly, systems-genetics analysis of a segregating *Eucalyptus* hybrid population integrated variation in transcripts, proteins, and metabolites with complex biomass-related traits. The analysis identified biologically meaningful gene sets and molecular networks associated with wood and biomass accumulation, demonstrating the potential of multi-omics approaches to connect molecular variation with quantitative phenotypes [80,81,83].

The integration of these datasets can be conceptualised as a progression from genotype to phenotype. Genomic information can first be used to identify variants, QTLs, or genomic regions associated with a target trait [70]. Transcriptomic data can then determine whether genes within these regions are differentially expressed or form relevant co-expression networks [80]. Proteomic data can provide evidence that changes at the transcript level are translated into differences in proteins or enzymes, while metabolomic profiles can determine whether these molecular changes are accompanied by alterations in the corresponding biochemical pathways. When these molecular patterns are consistently associated with phenotypic variation across genetically related individuals and environments, candidate genes and pathways become stronger targets for functional validation and breeding.

For secondary metabolism, this integration is particularly valuable because metabolite accumulation is controlled through interconnected biosynthetic pathways rather than by individual genes acting independently. For example, variation in lignin or phenylpropanoid-related traits may involve differences in the expression of pathway genes, abundance or activity of the corresponding enzymes, and the accumulation of downstream metabolites. A combined transcriptome–proteome–metabolome analysis can therefore distinguish between a gene that is merely differentially expressed and one that is associated with measurable changes in protein abundance and metabolic flux. Such information can improve the prioritisation of candidate genes for functional studies and potentially identify metabolic biomarkers associated with wood quality, defence, or stress adaptation.

Multi-omics integration can also contribute to the analysis of complex quantitative traits in *Eucalyptus* hybrids. Hybrid performance frequently reflects interactions among parental genomes and may involve changes in gene expression, regulatory networks, protein abundance, and metabolism that are not readily predicted from single-marker associations. Comparing contrasting hybrids, parents, and segregating populations across common environments can help identify molecular signatures associated with heterosis, growth, wood quality, and stress responses. Integration with phenotypic measurements and genomic prediction may ultimately allow molecular information to be used not only to explain trait variation but also to improve early selection of superior breeding material.

Several analytical approaches can be used to integrate multi-omics datasets, including correlation analysis, co-expression networks, pathway enrichment, network-based systems genetics, multivariate statistics, and machine-learning approaches. Rather than analysing each omics layer independently, these methods can identify relationships among genetic variants, transcripts, proteins, metabolites, and phenotypic measurements. Multi-omics integration is particularly informative when samples are collected from the same genotypes, tissues, developmental stages, and environmental conditions, because this reduces ambiguity when associations are interpreted across molecular layers.

Despite its potential, multi-omics integration presents important practical challenges for forest-tree breeding. The different datasets vary in scale, technical variation, detection limits, missing values, and statistical distributions. Sampling time and tissue type are also critical because transcript, protein, and metabolite profiles can change rapidly with developmental stage and environmental conditions. In addition, generating all molecular datasets for large breeding populations can be expensive. Consequently, comprehensive multi-omics profiling may initially be most appropriate for discovery populations or strategically selected contrasting genotypes, followed by validation of informative biomarkers or candidate genes in larger breeding populations.

The integration of multi-omics data should therefore complement rather than replace conventional phenotyping and genomic selection [80]. A practical breeding framework could use genome-wide markers to predict breeding values at the population level, while multi-omics analyses are applied to selected individuals or populations to clarify the biological mechanisms underlying target traits and identify candidate biomarkers. Candidate genes emerging from this process can subsequently be evaluated using molecular interaction assays, gene-expression studies, transgenic approaches, or genome editing before markers or genomic information are incorporated into breeding pipelines.

Recent developments in *Eucalyptus* multi-omics resources further support this direction. The EucaMOD platform, for example, integrates genomic, comparative genomic, pan-proteomic, transcriptomic, epigenomic, and variation datasets across multiple *Eucalyptus* species, providing a resource for functional genomics and molecular breeding [81]. However, metabolomic data remain less comprehensively integrated into such resources, indicating an important area for future development. The continued expansion of matched genomic, transcriptomic, proteomic, and metabolomic datasets, together with improved statistical and computational methods, could facilitate a systems-level understanding of complex traits in *Eucalyptus* and improve the identification and validation of molecular targets for breeding.

NGS provides the foundation for genome-wide discovery, whereas multi-omics integration extends this information towards biological interpretation and functional validation [70]. Combining genomics with transcriptomics, proteomics, and GC-MS-based metabolomics can help connect genetic variation with gene regulation, protein function, metabolic activity, and ultimately phenotype. For *Eucalyptus* breeding, this integrated approach is particularly promising for complex traits such as wood formation, secondary metabolism, growth, stress tolerance, and disease resistance, although its routine application to large-scale breeding populations will depend on reductions in analytical costs, standardisation of sampling and data processing, and validation across genetic backgrounds and environments.

#### 8.2.8. Genetic Engineering and Genome Editing

Genome editing is an emerging technology with considerable potential for *Eucalyptus* improvement because it enables targeted modification of genes associated with economically important traits. Unlike conventional breeding, which generally relies on repeated crossing, recombination, and selection across generations, genome editing can introduce specific genetic changes into elite breeding material while potentially retaining much of its existing genetic background [84]. However, the practical application of genome editing in *Eucalyptus* remains largely at the research and development stage, and its contribution to operational breeding will depend on successful transformation and regeneration, trait validation, stable inheritance, and appropriate regulatory approval.

Among genome-editing technologies, CRISPR-Cas systems have received considerable attention because of their versatility and ability to target specific genomic sequences. In *Eucalyptus*, genome-editing approaches have potential applications in traits relevant to plantation productivity and resilience, including disease resistance, wood properties, biomass production, and tolerance to abiotic stresses [85]. Genome editing can differ from conventional transgenic genetic engineering in that some editing strategies can generate targeted sequence changes without necessarily introducing a stable foreign DNA sequence into the final plant. Nevertheless, the molecular characteristics and regulatory status of the resulting genotype depend on the editing strategy and the applicable regulatory framework [86].

Technical and biological constraints remain important barriers to the wider application of genome editing in *Eucalyptus*. These include genotype-dependent transformation efficiency, regeneration of edited plants, potential unintended genetic changes, validation of edited phenotypes, stable inheritance, and the relatively long generation time associated with perennial forest trees. In addition, the testing, environmental release, and commercial deployment of genome-edited *Eucalyptus* must comply with the regulatory requirements of the relevant jurisdiction. Regulatory approaches differ among countries and may depend on the type and characteristics of the genetic modification rather than simply on whether genome editing was used. Environmental and biosafety considerations are particularly relevant for plantation trees because of their perennial nature, reproductive biology, potential pollen-mediated gene flow, and interactions with surrounding ecosystems.

Therefore, genome editing should be considered a complementary precision-breeding technology rather than a replacement for conventional breeding. Its greatest potential may arise when targeted genetic modification is integrated with genetic-resource management, parent selection, genomic characterization, controlled hybridisation, phenotypic evaluation, and clonal testing. Such integration could allow validated genomic targets to be incorporated into elite breeding material while retaining the broader breeding pipeline required to evaluate agronomic performance, stability, environmental adaptation, and regulatory suitability before deployment.

##### CRISPR-Cas9 and Precision Genome Editing

CRISPR-Cas9 has expanded the potential for precise genetic modification in *Eucalyptus*, but its application to complex stress-tolerance traits may extend beyond single-gene knockouts [87]. Stress responses involve interconnected networks of transcription factors, hormone signalling, protein turnover, ion homeostasis, and redox regulation; therefore, targeting regulatory components or cis-regulatory regions may provide more effective control of complex phenotypes. Protein ubiquitination and proteasomal degradation are particularly relevant because E3 ubiquitin ligases, ubiquitin-conjugating enzymes, deubiquitinating enzymes, and their target proteins regulate the stability of stress-responsive regulators [88]. Candidate components identified through genomic, transcriptomic, or proteomic analyses could therefore be investigated as potential CRISPR targets [82,83]. Similarly, editing transcription factors or their binding sites could modify groups of downstream stress-responsive genes, while promoter editing could fine-tune gene expression rather than completely eliminate gene function. These approaches may help improve responses to drought, salinity, temperature, and other stresses while reducing potential effects on normal growth and wood formation [87].

For complex and polygenic traits, multiplex CRISPR approaches could simultaneously modify functionally related genes or regulatory regions within a stress-response pathway. Integration with QTL, GWAS, transcriptomic, proteomic, and metabolomic information could help identify regulatory nodes most suitable for targeted editing and subsequent physiological and molecular validation. However, network-level genome editing in *Eucalyptus* remains largely prospective, and disruption of highly connected regulatory pathways may result in pleiotropic effects, off-target changes, or reductions in growth and wood quality [48]. CRISPR is therefore more appropriately viewed as complementary to genomic selection: genomic selection can capture the effects of many small-effect loci, whereas targeted genome editing can provide a means of testing and manipulating biologically important regulatory genes or pathways. Future applications should consequently prioritise functionally validated targets and evaluate edited material across genetic backgrounds and environments before incorporation into breeding programmes.

#### 8.2.9. Trait-Associated Genes and Quantitative Trait Loci in *Eucalyptus*

The identification of trait-associated genes and quantitative trait loci (QTLs) has improved understanding of the genetic basis of economically important traits in *Eucalyptus*. Rather than serving only as genomic landmarks, these discoveries provide a framework for connecting phenotypic variation with genomic regions and candidate genes. QTL mapping, genome-wide association studies (GWAS), high-throughput sequencing, and genomic prediction can therefore complement conventional phenotypic selection and potentially increase selection efficiency in breeding programmes [9,64].

Research over the past two decades has demonstrated that many commercially important *Eucalyptus* traits, including growth, wood density, fibre characteristics, rooting ability, and resistance or tolerance to environmental stresses, are quantitatively inherited and influenced by multiple loci [89]. This has shifted breeding strategies away from the expectation that complex traits are controlled by single major genes and towards approaches that capture the combined effects of multiple genomic regions. However, the usefulness of individual QTLs may be population- and environment-dependent, and their validation across pedigrees and environments remains important before deployment in operational breeding programmes.

Wood formation has been particularly well studied because of its direct importance to pulp yield, timber quality, and bioenergy production. Genomic studies have identified loci and candidate genes associated with cellulose biosynthesis, lignin deposition, fibre development, and wood density, providing resources for marker development, genomic selection, and functional studies [12]. Growth-related genomic studies have similarly contributed to understanding variation in tree height, stem diameter, and biomass accumulation. These resources have supported the development of genomic approaches for selecting high-performing *Eucalyptus* breeding material, although the predictive value of markers can vary among pedigrees and breeding populations.

Environmental adaptation is receiving increasing attention as climate variability intensifies. Genomic and physiological studies have investigated drought-related traits, cold responses, stomatal characteristics, and other adaptive phenotypes in *Eucalyptus*. QTL mapping has, for example, been applied to stomatal traits in interspecific *Eucalyptus* hybrids, demonstrating the feasibility of investigating adaptive physiological traits within hybrid breeding populations [9]. In drought-tolerant and drought-sensitive *Eucalyptus camaldulensis* × *E. urophylla* hybrid clones, differential expression of genes involved in ABA biosynthesis and signalling, including EgrNCED3 and EgrPYR1, together with EgrDREB2.5 and EgrCDPK26, has provided molecular evidence for contrasting drought-response mechanisms [90].

Salinity tolerance is another emerging target for genomic and molecular investigation. Physiological screening has demonstrated substantial variation among *Eucalyptus* clones in photosynthetic performance, ion regulation, biochemical responses, and growth under saline conditions [53]. At the molecular level, salt-responsive genes and regulatory networks have been identified in *Eucalyptus*, including transcription factors belonging to the FAR1/FHY3, HD-Zip, NF-YB, and PHR families [53,54,55,56,57,58,90]. More recently, comparative transcriptomic analysis of contrasting *Eucalyptus* clones exposed to prolonged salinity identified coordinated differences in genes associated with ABA and ethylene signalling, MAPK cascades, antioxidant defence, carbohydrate metabolism, protein folding, and osmotic and cell-wall responses [54,56]. These studies provide candidate genes and pathways that could ultimately support marker development and genomic prediction for salinity tolerance.

Experimental molecular breeding has provided additional evidence that manipulating stress-response genes can alter salinity tolerance in *Eucalyptus*. Transgenic *E. camaldulensis* expressing McRBP, an RNA-binding protein gene from the halophyte Mesembryanthemum crystallinum, showed improved performance under both severe and prolonged moderate salinity [90]. Similarly, expression of the codA gene in *E. globulus* increased glycinebetaine accumulation and improved salt-stress performance [58]. These experiments provide functional evidence for candidate mechanisms but remain proof-of-concept studies rather than established genomic-selection or marker-assisted breeding strategies for commercial salt-tolerant hybrids.

Recent research has also expanded the range of candidate genes that may contribute to abiotic-stress adaptation. For example, *Eucalyptus grandis* genes encoding DREB transcription factors have been investigated for responses to drought, cold, and salinity, while recent functional work on EgrDREB3 has demonstrated enhanced low-temperature and salinity resilience when the gene is expressed in Arabidopsis [91]. Because the latter evidence was generated in a heterologous model rather than *Eucalyptus* itself, it should be regarded as functional support for candidate-gene activity rather than direct evidence of an established *Eucalyptus* breeding application.

Trait-associated genes and QTLs provide increasingly valuable genomic resources for *Eucalyptus* breeding, but their application differs among traits. Genomic resources for growth and wood properties are considerably more developed than those for saline-alkaline tolerance. For salinity, current evidence is strongest for physiological screening, transcriptomic characterisation, candidate-gene identification, and experimental functional validation. Future breeding programmes could integrate these resources with controlled hybridisation, high-throughput genotyping, genomic prediction, and multi-environment evaluation to identify and validate alleles contributing to salt tolerance in *Eucalyptus* hybrids. Such integration will be particularly important because salinity tolerance is a complex phenotype influenced by genotype, developmental stage, stress intensity, and genotype-by-environment interactions.

Identification of candidate genes through QTL mapping, GWAS, transcriptomics, or comparative genomic analysis represents an important step in understanding complex traits, but genomic association alone does not establish gene function or causality. Functional validation is therefore an important link between genomic discovery and breeding application. Molecular interaction assays can help determine whether candidate proteins participate in the regulatory networks predicted from genomic and transcriptomic analyses. Yeast Two-Hybrid (Y2H) assays can be used to test protein–protein interactions, whereas Bimolecular Fluorescence Complementation (BiFC) can provide evidence of protein interaction in living cells and information on subcellular localisation [76,77]. Co-Immunoprecipitation (Co-IP) can provide additional evidence for protein associations under comparatively native cellular conditions [78].

For candidate transcription factors identified in *Eucalyptus*, Electrophoretic Mobility Shift Assay (EMSA) can be particularly informative because it tests whether a candidate protein directly binds a defined DNA sequence or promoter element [79]. This approach can help distinguish direct regulatory relationships from correlations identified through transcriptomic studies. For example, a candidate transcription factor identified as salt-responsive could be investigated using EMSA to determine whether it binds promoter regions of downstream genes involved in ion transport, osmotic adjustment, antioxidant defence, or stress signalling [77,79].

These techniques are most informative when combined with complementary functional approaches, including gene-expression analysis, promoter-reporter assays, heterologous expression, gene overexpression or silencing, and genome editing. Such combinations can provide evidence at different levels, from physical molecular interaction and DNA binding to changes in gene expression and ultimately the target phenotype. Nevertheless, the application of these approaches in *Eucalyptus* remains more developed for some biological processes than for complex breeding traits such as saline-alkaline tolerance. Therefore, Y2H, BiFC, Co-IP, and EMSA should be viewed as valuable functional-validation tools with considerable application potential rather than as universally established components of operational *Eucalyptus* breeding programmes.

### 8.3. Epigenetic Regulation and Chromatin-Mediated Stress Responses

DNA sequence variation alone does not fully explain differences in stress responses among *Eucalyptus* genotypes. Epigenetic mechanisms, including DNA methylation, histone modifications, chromatin accessibility, and nucleosome organisation, provide an additional layer of regulation that can alter the expression of genes involved in growth, secondary metabolism, and responses to drought, salinity, temperature, and other environmental stresses [49]. Chromatin immunoprecipitation (ChIP), particularly ChIP-seq, can be used to identify histone modifications or transcription-factor occupancy at regulatory regions, thereby linking chromatin state with stress-responsive gene expression [92]. Integrating ChIP-based information with QTL, GWAS, transcriptomic, proteomic, and metabolomic data could help distinguish sequence variation from environmentally induced regulatory effects and identify candidate genes and regulatory regions underlying stress adaptation in *Eucalyptus* hybrids. Such information may also improve understanding of genotype-by-environment interactions and provide targets for functional validation and future molecular breeding. However, because some epigenetic changes may be transient or environmentally induced rather than stably inherited, their potential use as selection criteria requires validation across genotypes, environments, developmental stages, and generations. Epigenomic approaches should therefore be considered complementary to, rather than a replacement for, DNA-based genomic selection.

### 8.4. An Integrated Pipeline for Eucalyptus Hybrid Breeding

The development of improved *Eucalyptus* hybrids is not dependent on a single breeding technique but involves a sequence of interconnected decisions extending from the identification of genetically valuable parents to the deployment of tested clones [6]. An effective hybrid-breeding programme integrates genetic-resource management, parent selection, reproductive biology, controlled crossing, progeny evaluation, molecular characterization, clonal testing, and multi-environment evaluation [68]. The integration of these components is particularly important in *Eucalyptus*, where high levels of heterozygosity, substantial genetic variation among and within species, asynchronous flowering, reproductive barriers, and genotype × environment interactions can influence the efficiency of hybrid development.

Suitable parent material is identified and characterized, and this is the start of the breeding process. Genetic resource collections, breeding archives, provenance trials and operational breeding populations represent sources of genetic diversity as shown in Figure 1. Parents could be selected based on phenotypic performance for growth, wood properties, disease resistance, drought adaptation or other target traits, while molecular markers and genomic information can provide additional information on genetic relationships, population structure and breeding value. The combination of phenotypic and genomic information can help breeders to choose complementary parents and to reduce the risk of crossing closely related materials.

Another important part of hybrid production after parent selection is reproductive biology. The success of controlled pollination may depend on flowering synchrony, flowering intensity, pollen availability, pollen viability, stigma receptivity, ploidy and cross-compatibility [5]. Hence, controlled pollination techniques such as conventional controlled pollination and reproductive manipulation approaches are used to control pollen transfer and enhance the chances of obtaining the desired hybrid combinations. Pollen storage and viability can be evaluated to further extend the crossing window when parental flowering is not synchronous [28]. Such reproductive technologies are the tangible connection between genetic selection of parents and the production of hybrid offspring.

The resulting progeny are then evaluated for hybrid fidelity, survival, growth, disease response, wood properties and other breeding objectives. At this stage it is possible to add molecular markers for verification of parentage and hybrid identity, and to estimate genetic relationships and genomic breeding values using genome-wide markers. Quantitative trait locus mapping and genome-wide association studies provide complementary approaches for identifying genomic regions associated with traits of interest, whereas genomic selection can use genome-wide marker information to predict the breeding value of individuals before traits can be measured at maturity [6,9,12]. These approaches can therefore shorten breeding cycles and improve the efficiency of selecting individuals for subsequent testing.

Promising hybrid progeny are commonly advanced through clonal propagation and replicated field testing. Clonal testing enables the evaluation of genetically identical material across sites and environments and can reveal genotype × environment interactions that are important for commercial deployment. Selection at this stage combines growth, wood quality, disease resistance, adaptation, and stability with practical considerations such as propagation ability and operational performance. Elite clones that consistently demonstrate superior performance can subsequently be multiplied and deployed commercially.

Thus, *Eucalyptus* hybrid breeding can be viewed as an iterative pipeline in which different technologies address different stages of the breeding process: genetic resources provide diversity; phenotypic and genomic information guide parent selection; reproductive technologies enable targeted hybridisation; molecular tools verify and characterize progeny; phenotypic and genomic selection identify superior individuals; and clonal and multi-environment testing determine whether selected hybrids are sufficiently stable and productive for deployment. The integration of these approaches is more informative than considering each technology independently because the value of a breeding technology ultimately depends on how effectively it improves decisions at subsequent stages of the breeding cycle.

### 8.5. Conventional and Molecular Approaches in Eucalyptus Breeding and Hybrid Development

The traditional selection, pollination, and molecular approaches in *Eucalyptus* improvement are summarised in Figure 1. Breeding *Eucalyptus* has progressed from classical phenotypic selection to include molecular and genomic technologies that improve the efficiency, accuracy, and rate of genetic gain. Conventional breeding is the main route of *Eucalyptus* improvement, exploiting naturally occurring genetic variation through selection of superior individuals and controlled hybridisation. Nevertheless, conventional breeding alone has had slow progress due to long generation time in forest trees, quantitative inheritance of many economically important traits, and large environmental influences on phenotype [6,63]. Hence, integration of molecular breeding methods complements conventional breeding procedures by providing additional genetic information for effective selection [68].

## 9. Conventional Breeding Approaches in *Eucalyptus*

Conventional breeding methods have traditionally been used for the genetic improvement of *Eucalyptus* species [6]. These approaches usually favour better individuals with desirable phenotypic traits, such as fast growth, disease resistance, and high wood quality. Controlled crosses have been made among selected complementary genotypes, and desirable combinations of traits have been selected in successive clonal progeny generations. The process is based on natural variation in a species’ genetics and phenotypic selection. This causes slow improvements over time.

Traditional breeding has its limitations, such as a slow rate of genetic gains and difficulty in selecting for complex traits, particularly those controlled by multiple genes [63]. In *Eucalyptus* breeding programs, the gains in selection for wood quality or disease resistance can be slow. Therefore, there is an increasing need to combine biochemical and molecular tools with conventional methods to speed up the development of better genotypes [68].

### 9.1. Pollination Methods in Eucalyptus

Hybrid breeding in *Eucalyptus* requires efficient cross-pollination techniques to combine desirable traits from genetically diverse parents and to exploit hybrid vigour. This process depends on controlled pollination, which ensures seed production from defined parental combinations, to increase the precision of genetic evaluation and the efficiency of breeding programmes [4,5,28,29]. With the change in breeding objectives from small-scale genetic studies to the commercial production of hybrid seed, pollination methods have been modified to balance genetic precision with operational efficiency.

*Eucalyptus* breeding employs a range of pollination methods, from natural open pollination to controlled methods, such as conventional controlled crossing, One-Stop Pollination (OSP), and Artificially Induced Protogyny (AIP). The approaches differ in the degree of parental control, labour requirements, scalability, and suitability for specific breeding objectives (Figure 1). Thus, the choice of a proper pollination strategy depends on the main goal: genetic analysis, hybrid development, or operational seed production.

#### 9.1.1. Open Pollination/Poly Crosses

Open pollination is the natural reproductive system of *Eucalyptus*, involving pollen transfer mediated by insect pollinators and other environmental vectors, without any human intervention. This method gives little control over pollen parentage but is useful for producing half-sib families for progeny testing, seed orchard management, and studies of genetic diversity [4]. Open pollination is usually unsuitable for hybrid breeding programmes that require accurate pedigree records, as parentage cannot be guaranteed. However, it is a useful and economical method of preserving wide genetic diversity and developing breeding populations from which superior individuals may later be selected.

#### 9.1.2. Controlled Conventional Crosses

*Eucalyptus* has traditionally been considered a model for controlled crossing to generate genetically verified full-sib families. This procedure includes flower emasculation, application of selected male pollen under controlled conditions, and subsequent isolation of pollinated flowers to avoid contamination [28,29]. Such control of parental selection makes traditional/conventional crossing an essential tool for genetic studies, quantitative trait analysis, and early-stage breeding, with pedigree accuracy being critical.

This method is precise but labour-intensive; multiple field visits and reliance on synchronised flowering between parents are limitations of conventional crossing. These operational constraints diminish its suitability for large-scale commercial hybrid seed production and have led to the development of more efficient pollination systems, such as One-Stop pollination [28].

#### 9.1.3. One-Stop Pollination (OSP)

One-Stop Pollination was developed to enhance the operational efficiency of controlled crossing by combining emasculation, pollination, and flower isolation into one field operation [29]. OSPs reduce labour costs substantially by reducing the number of visits per cross while maintaining high levels of genetic control. Horsley [28] also showed that OSP had minimum self-pollen contamination in small-flowered *Eucalyptus*, corroborating its appropriateness for hybrid seed production.

OSP improves operational efficiency over conventional crossing, but its success is dependent on the developmental stage of the flower and environmental conditions. Furthermore, some studies have reported a lower seed set, relative to conventional methods, which suggests that efficiency gains in labour might sometimes come at the expense of a lower reproductive output [28]. These results suggest that OSP does not fully substitute conventional crossing but rather involves a compromise between genetic resolution and operational scalability.

#### 9.1.4. Artificially Induced Protogyny (AIP)

One of the most important innovations in *Eucalyptus* controlled pollination is Artificially Induced Protogyny. Unlike traditional methods that involve complete emasculation, AIP manipulates floral development by removing the tip of the operculum and part of the style to expose receptive stigmas before pollen release, thus converting the naturally protandrous flower into a functionally protogynous one [5,28]. This simple intervention eliminates several labour-intensive steps, while still maintaining acceptable levels of crossing accuracy.

AIP has been shown to greatly improve operational efficiencies. Assis et al. [5] reported seed yields like conventional pollination, but with productivity three to eighteen times higher per operator hour. Horsley [28] came to the same conclusions, pointing out the suitability of AIP for small-flowered *Eucalyptus* species where traditional emasculation is difficult and expensive. At advanced developmental stages of flowers, slightly increased risks of self-pollination have been reported, but high genetic fidelity of AIP-derived progeny has generally been confirmed by molecular verification. In conclusion, these studies indicate that AIP is one of the most practical approaches for large-scale operational breeding and commercial hybrid seed production.

### 9.2. Comparative Perspectives and Future Directions

The transition from conventional controlled crossing to OSP and AIP represents the wider evolution of *Eucalyptus* breeding towards strategies that maximise genetic gain and operational efficiency [28]. Traditional crossing still plays an important role when pedigree accuracy is critical. OSP, particularly AIP, offers scalable options that can underpin commercial hybrid breeding programmes. Open pollination is important in maintaining genetic diversity and establishing breeding populations but is not suitable where controlled parentage is required. Table 3 summarises these methods.

Despite considerable progress, no single pollination method fulfils all breeding goals. The effectiveness of the techniques varies with species, flowering characteristics, pollen viability, environmental conditions, and the requirements of the breeding programme [4]. Future research should therefore focus on extending the present work to improve pollination protocols for different *Eucalyptus* genotypes, to combine molecular parentage verification with operational breeding, and to develop automated or semi-automated pollination technologies that can further reduce labour costs while maintaining genetic integrity. Such advances will be essential to meet the growing demand for improved hybrid germplasm in commercial forestry.

## 10. Perspective and Conclusions

The review synthesizes the integrative approaches in breeding of *Eucalyptus* hybrids with emphasis on genetic resource management. Traditional and molecular breeding tools for *Eucalyptus* improvement programs have evolved from phenotype-driven selection to data-informed, genome-enabled approaches, and from germplasm collection to the application of genomics and precision breeding. Phenotypic selection and controlled crosses, for example, are still part of the whole breeding pipeline, especially in early stages of parental selection and trait validation. However, the limitations of molecular breeding technologies have been gradually overcome, particularly in polygenic traits, long generation times, and environmental variability. Genotyping, QTL mapping, and next-generation sequencing technologies have improved the accuracy and speed of the selection process to allow breeders to develop genotypes that meet the twin challenges of productivity and adaptability. The results of these integrated efforts include the development and implementation of modern hybrid genotypes such as *E. grandis* × *E. urophylla*, *E. grandis* × *E. nitens*, *E. camaldulensis* × *E. globulus*, and *E. urophylla* × *E. pellita*.

Modern hybrids are the basis of commercial forestry globally, including South America, Southeast Asia, and sub-Saharan Africa. Breeding gains have improved the economic value of plantations, supported rural livelihoods, agroforestry systems, and climate-resilient landscapes. Future breeding of *Eucalyptus* hybrids will use genome editing technologies such as CRISPR-Cas9, high-throughput phenotyping, machine learning-based trait prediction, and increased access to genomic databases. These tools promise shorter breeding times, greater selection accuracy, and the tailoring of genotypes to specific ecological and industrial environments. But it will face challenges, including the high cost of infrastructure, regulatory uncertainty around GMOs, and capacity-building in developing regions.

Artificial intelligence (AI) and machine learning (ML) provide further opportunities to address the complexity of genotype-by-environment (G×E) interactions in *Eucalyptus* breeding by integrating multidimensional environmental and genomic datasets. Future breeding programmes could combine high-density SNP genotypes with environmental variables such as temperature, rainfall, humidity, solar radiation, drought indices, and growing-season conditions, together with soil characteristics including pH, texture, organic matter, nutrient availability, water-holding capacity, and soil moisture. These datasets could be linked with repeated phenotypic measurements of growth, wood quality, disease response, water-use efficiency, and survival across multiple locations and seasons. Machine-learning algorithms, including random forests, gradient-boosting methods, support vector machines, and deep-learning approaches, could then be trained to identify nonlinear relationships and interactions between genotype, environment, and phenotype that may be difficult to detect using conventional statistical approaches. The resulting models could be used to predict the performance and stability of candidate genotypes under current and projected environmental conditions, identify environments in which specific genotypes are likely to perform best, and support genomic selection for target production zones. Integration with spatial environmental datasets and climate projections could further enable breeders to anticipate future G×E patterns and prioritise genotypes with broad or specific adaptation to drought, heat, soil constraints, and other environmental stresses. However, AI-based prediction should be supported by sufficiently large and representative training datasets, independent validation across environments and breeding populations, and interpretable models to ensure that predicted genotype–environment relationships are biologically meaningful. Such integration could transform G×E analysis from a largely retrospective evaluation into a predictive component of *Eucalyptus* breeding and accelerate the development of climate-resilient, site-specific next-generation forest varieties.

Finally, there is increasing recognition that breeding success must be measured not only in terms of productivity but also in terms of sustainability indicators, such as water-use efficiency, reduced production inputs, carbon sequestration, and biodiversity conservation. A modern breeding program thus needs to be multidisciplinary, balancing commercial objectives with ecological stewardship and socio-economic equity. *Eucalyptus* hybrid breeding is a model of applied genetic science that delivers economic and environmental value as the global forestry sector transitions to climate-smart, circular bioeconomies. The future of the field will depend on technical innovation, collaborative research, policy support, and equitable access to breeding technologies. The review may serve as a framework to guide *Eucalyptus* breeding and genetics, aiming to develop next-generation hybrids that adapt to environmental changes and meet market demands.

## Figures and Tables

**Table 2:** Genetic resource collections and genomic databases relevant to *Eucalyptus* breeding and conservation.

Genetic Resource/Database	Location/Access	Function and Relevance to *Eucalyptus*	References
Australian Tree Seed Centre (ATSC), CSIRO	Canberra, Australia; physical collection and online access	National tree-seed collection maintained by CSIRO. The collection contains seed from Australian tree species, including a large representation of *Eucalyptus* and *Corymbia*, and supports genetic-resource conservation, research, seed supply and breeding.	[35]
Embrapa Florestas *Eucalyptus* and *Corymbia* Seed Bank	Colombo, Paraná, Brazil	Ex situ conservation of *Eucalyptus* and *Corymbia* germplasm. The collection contains approximately 3000 seed samples representing 68 *Eucalyptus* and *Corymbia* species and includes provenances and progenies originating primarily from natural populations.	[36]
EucGenIE	Online	*Eucalyptus grandis*-focused genomic and transcriptomic resource integrating genome and gene-expression information for functional genomics and comparative studies.	[37,38]
B EUCANEXT	Online; Brazil	Genomic and transcriptomic resource containing genome, transcriptome and expressed-sequence-tag information for *Eucalyptus* species.	[39,40]
EucaMOD (Eucalyptus multi-omics database)	Online; Guangxi University, China	Integrated multi-omics database for *Eucalyptus*. It incorporates genome, comparative-genome, pan-proteome, transcriptome, epigenome and variation data and provides tools for functional genomics and molecular breeding.	[41]

**Table 3:** Comparative analysis of controlled pollination techniques.

Method	Precision	Scalability	Labour Requirements	Application	References
Open Pollination	Low	High	Very low	Genetic diversity assessments, seed orchards	[28,29]
Controlled crosses	Very high	Low	High	Early-stage breeding, genetic studies	[29]
One-Stop Pollination (OSP)	High	Medium-High	Moderate	Commercial hybrid seed production	[28]
Artificially induced protogyny (AIP)	Moderate	High	Very low	Operational breeding, seed production	[5]

## Data Availability

No new data were created or analyzed in this study. Data sharing is not applicable to this article.
